# Carbon Nanodots as Functional Excipient to Develop Highly Stable and Smart PLGA Nanoparticles Useful in Cancer Theranostics

**DOI:** 10.3390/pharmaceutics12111012

**Published:** 2020-10-23

**Authors:** Nicolò Mauro, Mara Andrea Utzeri, Salvatore Emanuele Drago, Gianpiero Buscarino, Gennara Cavallaro, Gaetano Giammona

**Affiliations:** 1 Lab of Biocompatible Polymers, Department of Biological, Chemical and Pharmaceutical Sciences and Technologies (STEBICEF), University of Palermo, via Archirafi 32, 90123 Palermo, Italy; maraandrea.utzeri@unipa.it (M.A.U.); salvatoreemanuele.drago@unipa.it (S.E.D.); gianpiero.buscarino@unipa.it (G.B.); gennara.cavallaro@unipa.it (G.C.); gaetano.giammona@unipa.it (G.G.); 2 Fondazione Umberto Veronesi, Piazza Velasca 5, 20122 Milano, Italy; 3 Department of Physics and Chemistry (DiFC), University of Palermo, via Archirafi 36, 90123 Palermo, Italy; 4 Institute of Biophysics at Palermo, Italian National Research Council, Via Ugo La Malfa 153, 90146 Palermo, Italy

**Keywords:** PLGA, carbon nanodots, cancer theranostics, photothermal therapy, hybrid nanoparticles, imaging

## Abstract

Theranostic systems have attracted considerable attention for their multifunctional approach to cancer. Among these, carbon nanodots (CDs) emerged as luminescent nanomaterials due to their exceptional chemical properties, synthetic ease, biocompatibility, and for their photothermal and fluorescent properties useful in cancer photothermal therapy. However, premature renal excretion due to the small size of these particles limits their biomedical application. To overcome these limitations, here, hybrid poly(lactic-*co*-glycolic acid) (PLGA-CDs) nanoparticles with suitable size distribution and stability have been developed. CDs were decisive in the preparation of polymeric nanoparticles, not only conferring them photothermal and fluorescent properties, needed in theranostics, but also having a strategic role in the stabilization of the system in aqueous media. In fact, CDs provide stable PLGA-based nanoparticles in aqueous media and sufficient cryoprotection in combination with 1% PVP. While PLGA nanoparticles required at least 5% of sucrose. Comparing nanosystems with different CDs content, it is also evident how these positively impinge on the loading and release of the drug, favoring high drug loading (~4.5%) and a sustained drug release over 48 h. The therapeutic and imaging potentials were finally confirmed through in vitro studies on a breast cancer cell line (MDA-MB-231) using fluorescence imaging and the MTS cell viability assay.

## 1. Introduction

In the era of precision medicine, theranostic nanomaterials represent a new valid approach to drug development through a strategic combination of tumor diagnosis, monitoring and therapy in a single targeted nanoplatform, resulting in overcoming severe limitations of conventional chemotherapy which often lead to the failure of treatment and the development of multidrug resistance (MDR) [1,2]. One advanced application of theranostic nanomaterials is imaging-guided photothermal therapy (IG-PTT), a promising strategy which combines the use of near-infrared (NIR) laser photoabsorbers with contrast agent properties. While the image guidance provides a precisely localization of the tumor region, the biodistribution of the nanosystems and the monitoring of the treatment, the conversion of NIR light energy into heat over the physiological temperature range (41–45 °C) by photothermal agents induces selective thermal ablation of cancer cells and their hypersensitization towards xenobiotics [3,4,5]. In particular, fluorescence imaging (FLI) using red to NIR emitters is an attractive technique for cancer detection due to high light tissue penetration and negligible tissue auto-fluorescence, which allows to exploit elevated sensitivity and spatio-temporal resolution of contrast images [6]. Various nanomaterials with low toxicity, strong fluorescence and photothermal conversions property within the biological transparency window (620–1100 nm) have been proposed for IG-PTT application such as organic dyes, noble metal nanoparticles and carbon derivates. However, despite the higher thermo/photostability of noble metal nanoparticles (e.g., gold nanoparticles) against organic dyes, they are characterized by a low renal excretion with possible organ deposits and potential long-term toxicity which limit their clinical application [7,8,9].

Carbon nanodots (CDs) are an emerging ultrasmall zero-dimensional (0D) carbon nanomaterial which have attracted intensive interest for their multi-color emissions, tunable optical properties, excellent photo/thermostability and easy surface functionalization. Moreover, CDs are usually characterized by good biocompatibility and rapid excretion from the body via renal filtration thanks to extremely small size, in contrast with other carbon derivates such as carbon nanotubes and graphene oxide [7,10,11,12,13].

Size, shape and surface properties have a key role in the distribution of nanocarriers in the organism. These parameters can be refined to increase their presence in the bloodstream and improve targeting for a specific site [14,15]. According to the EPR effect, a size between 10–200 nm allows a preferential localization of nanocarriers in the tumor tissue [15,16]. Taking this in mind, in order to increase the circulation lifetime of CDs in the bloodstream, and therefore the accumulation of CDs in tumor tissue, one strategy could be the encapsulation of the CDs inside bioresorbable nanoparticles [17]. The most common materials for the preparation of bioresorbable nanoparticles are represented by polymers [18], and among them polyesters like polylactic acid (PLA), poly-ε-caprolactone (PCL) and poly-d,l-lactide-*co*-glycolide (PLGA) represent an important class of biodegradable polymers for the production of pharmaceutical formulations for drug delivery and tissue engineering applications [15,19,20,21,22]. In particular PLGA, an FDA approved copolymer, has received great attention for the development of drug delivery system because in the organism it is hydrolyzed releasing metabolites such as lactic acid and glycolic acid, which in the Krebs cycle are metabolized to H_2_O and CO_2_. For these reasons, the systemic toxicity associated with the use of PLGA is minimal [19]. PLGA-based nanocarriers allow an optimal bioavailability of the encapsulated drugs, reducing the phenomena of premature degradation in the biological environment [23], providing a release of drugs governed by degradation kinetics and targeted delivery [24] and facilitating penetration in intracellular compartment of the bioactive molecules thusreducing side effects [25]. However, in literature are reported opposite opinions regarding drug release because sometimes PLGA based nanosystems show a premature and/or initial burst drug release. Moreover, PLGA based nanostystems show also others limitations such as instability in the aqueous environment, relatively low drug loading capacity and inability to release the drug on demand in the site of action. In the light of these, the aim of the present work is the development of a stable theranostic hybrid nanosystem obtained by encapsulation of highly hydrophilic CDs in PLGA nanoparticle. The hybrid CDs-PLGA nanoparticles are used for the delivery of irinotecan towards breast cancer cells. Here, we focused our efforts on the formulation aspect of the nanosystem, evaluating how the amount of encapsulated CDs (1–20%) influences the technological and therapeutic properties of the system. Our results demonstrate that the increase in CDs content from 1 to 20% (on a weight basis) allow obtaining more stable nanosystems with satisfactory efficacy and diagnostic properties, as demonstrated by in vitro studies.

## 2. Materials and Methods

### 2.1. Materials

Urea (99%), citric acid (99.5%), N,N-dimethylformamide (DMF), ethanol absolute, Sephadex G15 and G25, dialysis tubing MWCO 2 kDa, poly (D,L lactide-*co*-glycolide) (PLGA) (lactide/glycolide ratio 50:50 Mw 7000–14,000), irinotecan hydrochloride, dimethyl sulfoxide (DMSO), phosphate buffer saline (PBS) were purchased from Sigma Aldrich (Milan, Italy) and used as received.

### 2.2. Cell Culture

MDA-MB-231 cell line was purchased from Sigma Aldrich (Milan, Italy) and cultured in supplemented Dulbecco’s Minimum Essential Medium (DMEM) supplemented with 10% fetal bovine serum (FBS, Euroclone, Milan, Italy), 1% of penicillin/streptomycin (10,000 U mL^−1^ and 10 mg mL^−1^ respectively, Euroclone, Milan, Italy) and 1% of L-glutamine (Euroclone, Milan, Italy), at 37 °C in 5% CO_2_ humidified atmosphere. Cell Titer 96 Aqueous One Solution Cell Proliferation assay (MTS solution) were purchased from Promega (Milan, Italy).

### 2.3. Synthesis of CDs

CDs were synthesized as mentioned in our previous work [26]. Briefly, carbon nanodots were prepared by dissolving citric acid (3 g) and urea (6 g) in anhydrous DMF (30 mL). The reaction was conducted in solvothermal condition at 160 °C for 4 h. The product was precipitated drop by drop in ethanol; the precipitate was collected by centrifuging at 10,000 rpm for 10 min and then it was dispersed in ultrapure water by sonicating. The water dispersion of CDs with different size was purified by size exclusion chromatography (SEC), combining three types of sephadex in serie (G25, G15, G10). Subsequently, only the fractions with good fluorescence emission in the red region and NIR-sensitive photothermal property was selected.

### 2.4. Size Distribution and Structural Characterization of CDs

The size distribution of CDs was evaluated by atomic force microscopy (AFM, Bruker FAST-SCAN) (Milan, Italy) in soft tapping mode. The sample (0.1 mg L^−1^, 10 μL) was deposited on a mica substrate and dried in vacuum (10 mbar).

The identification of the surface functional groups was conducted by FT-IR spectroscopy (Bruker Alpha II spectrometer) (Milan, Italy). The sample was prepared as pellets of KBr.

### 2.5. Preparation of PLGA-CDs Nanoparticles by Solvent Displacement Technique (Nanoprecipitation)

To prepare PLGA-CDs1% nanoparticles, 99 mg of PLGA and 1 mg of CDs were dispersed in 10 mL of DMSO. The organic mixture was put in a burette and added dropwise to 100 mL of distilled water under continuous stirring and it was left under stirring for 1 h. Then, the aqueous dispersion was filtered and dialyzed against water (Visking Dialysis Tubing 18/32″, 2000 molecular weight cut-offs) overnight. Subsequently, the content of dialysis was aliquoted to test the influence of different type and amount of cryoprotectant agent (sucrose 1%, sucrose 2%, sucrose 5% trealose 2%, PVP 10%, lactose 1%, lactose 2%, lactose 5%) on NPs aggregation pre- and post-freeze-drying.

In the same way, PLGA-CDs20% nanoparticles were prepared dissolving 80 mg of PLGA and 20 mg of CDs. Drug loaded nanoparticles were also prepared, adding 25 mg of irinotecane hydrochloride to the solutions described above.

### 2.6. Dynamic Light Scattering Measurements (DLS)

DLS measurements were performed on 1 mL of fresh sample prepared as described above and also on lyophilized samples at 25 °C using a Malvern Zetasizer NanoZS instrument (Rome, Italy) equipped with a 532 nm laser with a fixed scattering angle of 173°, and the Dispersion Technology Software 7.02 software (Malvern Panalytical ltd, Almelo, The Netherlands).

Zeta-potential measurements were performed by aqueous electrophoresis measurements, recorded at 25 °C using the same apparatus for the DLS measurement. The Zeta-potential values (mV) were calculated from electrophoretic mobility using the Smoluchowski relationship. All analyses were performed in triplicated.

### 2.7. Atomic Force Microscopy of the PLGA-CDs Nanoparticles (AFM)

The size distribution and morphology of the dried PLGA-CDs nanoparticles were assessed by Atomic Force Microscopy (AFM, Bruker FAST-SCAN) (Milan, Italy) in a soft tapping mode and using a triangular tip with an apical radius of about 5 nm. Each image was obtained with a resolution comparable to the tip radius. For the measurements a dilute dispersion of the samples was deposited on a mica substrate and dried under vacuum (10 mBar) before the observation.

### 2.8. Optical Characterization of the PLGA-CDs Nanoparticles

The optical absorption properties (220–700 nm range) of CDs and the hybrid PLGA-CDs nanoparticles were carried out by a double beam spectrophotometer (Shimadzu UV-2401PC) (Milan, Italy) in a 1 cm quartz cuvette. The emission spectra were recorded by a spectrofluorometer (Shimadzu RF-5301PC) (Milan, Italy) under the same excitation wavelength of 540 nm. The optical measurements were performed on diluted aqueous dispersions.

### 2.9. Evaluation of Photothermic Effect of PLGA-CDs Nanoparticles

The photothermic effect was evaluated by exciting an aqueous dispersion of either PLGA-CDs1% or PLGA-CDs20% (0.2 mL, 0.1 mg mL^−1^) with an 810 nm laser with the power fitted at 7 and 2.5 W cm^−2^, respectively. At fixed intervals the temperature of the dispersion was reported as a function of the exposure time. Equal volume of water was used as negative control.

### 2.10. Drug Loading and Release Kinetics of PLGA-CDs (PLGA-CDs1%@IT and PLGA-CDs20%@IT)

The drug payload was evaluated dissolving a known amount of PLGA-CDs@IT NPs (~5 mg) in 400 μL of acetonitrile under magnetic stirring for 2 h, filtering this solution through a syringe filter of 0.45 nm and adding methanol (400 μL) and PBS pH 3.5 (1.2 mL) in order to obtain the eluent. The amount of irinotecan loaded inside the PLGA-CDs@IT NPs was determined by HPLC analysis. Analyses were performed with a mobile phase constituted by a mixture of 0.03 N PBS pH 3.5/CH_3_CN/MeOH 60:20:20 and a flow rate of 1 mL min^−1^. The column was C6 phenyl by Phenomenex^®^ (Castel Maggiore, Italy) with the temperature settled at 25 °C, and the detection wavelength was 366 nm.

The obtained peak area was compared with a calibration curve obtained by plotting areas versus standard solution concentrations of IT in the range of 0.001–0.0001 mg/mL (y = 106872x, R^2^ = 0.9999). The drug loading obtained were 3.15 and 4.73% (*w*/*w*) for PLGA-CDs1%@IT NPs and PLGA-CDs20%@IT NPs, respectively. The encapsulation efficiency was 16 and 24%, respectively.

For drug release kinetic studies an amount of PLGA-CDs1%@IT NPs and PLGA-CDs20%@IT NPs corresponding to a drug concentration of 0.2 mg mL^−1^ was dispersed in PBS pH 7.4 (1 mL) and placed into a dialysis tubing (MWCO 2 kDa) against 9 mL of PBS at 37 °C under orbital stirring (100 rpm). At defined set times 0.2 mL of the external medium were withdrawn and replaced with equal volume of fresh medium up to 48 h. The photothermal triggered drug release was evaluated irradiating (before the dialysis process) the dispersion of either PLGA-CDs1%@IT NPs or PLGA-CDs20%@IT NPs in PBS pH 7.4 (0.2 mg mL^−1^) by 810 nm laser (GBox 15A/B by GIGA Laser, Wuhan, China) for 200 s at 7 W cm^−2^ and 100 s at 2.5 W cm^−2^, respectively. The amount of IT released was calculated by HPLC analysis as described for drug loading determination.

### 2.11. In Vitro Anticancer Activity of PLGA-CDs20%@IT Nanoparticles

The anticancer activity of the IT loaded PLGA-CDs20% NPs was evaluated in vitro on the MDA-MB-231 cell line. Cells were incubated for 24 h in DMEM at a density of 2 × 10^4^ cell per well. After this time, the medium was replaced with 0.2 mL of fresh medium containing the PLGA-CDs20%@IT nanoparticles at different concentrations corresponding to equivalent concentrations of irinotecan ranging from 5 to 150 μg mL^−1^. After 24 and 48 h of incubation, cell viability was assessed by the MTS assay. In particular, samples were taken away from wells and replaced by fresh medium (100 μL) and an MTS assay solution (20 μL) was added. Then, cells were incubated for additional 2 h at 37 °C before reading the absorbance at 492 nm. A solution of irinotecan hydrochloride at the same concentration was used as positive control, while the used cryoprotectant (PVP) at the corresponding concentrations was used as negative control.

In another experimental set the NIR-triggered photothermal ablation of cells was evaluated using an 810 nm laser diode for 100 s for well with the power fitted at 2.5 W cm^−2^. Cells were seeded in a 96-well plate at a density of 2 × 10^4^ cells per well (200 μL) and growth in DMEM. After this, cells were treated with PLGA-CDs20%@IT NPs (5–150 μg mL^−1^) for 24 or 48 h and then irradiated with the laser before making MTS assay as above described. The increasing in temperature inside each well owing to the photothermal effect was previously assessed by mimicking the same conditions tested in vitro and by irradiating the dispersions of the nanosystem in DMEM. The temperature inside wells was monitored by using a fiber optic (CEM^®^, Cologno al Serio, Italy).

### 2.12. The 2-D Cell Uptake of PLGA-CDs20%NPs

Cell uptake of PLGA-CDs1% NPs and PLGA-CDs20% NPs (0.5 mg mL^−1^) was evaluated by fluorescence microscopy (Axio Cam MRm, Zeiss, Oberkochen, Germany) on MDA-MB-231 after 4, 6 and 24 h of incubation. Cells were seeded in an 8-well plate at a density of 1 × 10^4^ cells per well (200 μL) and growth in DMEM. After 24 h the medium was replaced with equal volume of fresh medium containing the nanosystem (0.5 mg mL^−1^). After different incubation time, nuclei were stained with 4′,6-diamidino-2-phenylindole (DAPI) washing cells with DPBS pH 7.4, adding the DAPI solution (100 μL) and incubating cells for 10 min. Cells were then washed up with fresh DPBS (200 μL three times) and images were recorded by a fluorescence microscope using a Zeiss Axio Cam MRm (Zeiss AG, Oberkochen, Germany).

## 3. Results and Discussion

### 3.1. Preparation and Physicochemical Characterization of the Carbon Nanodots

**Figure 1:**
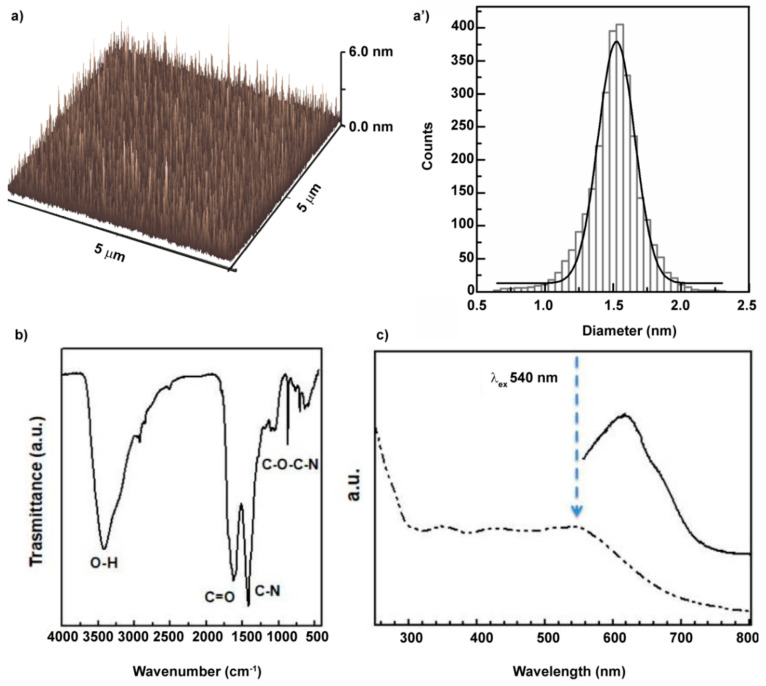
Chemical and physicochemical characterization of CDs. Size distribution of CDs by atomic force microscopy (**a**,**a’**). Surface characterization by FTIR analysis (**b**). Absorption UV/vis spectrum and emission spectra exited at 540 nm of aqueous dispersion of CDs (**c**).

Carbon nanodots (CDs) were prepared by solvothermal reaction from urea and citric acid in anhydrous DMF. This bottom-up synthetic way usually affords to a mixture of amorphous materials and crystalline nanoparticles with dimension within 1–10 nm [27]. Obviously, this heterogeneous mixture impinges on the optical properties and on the process of developing effective nanosystems for biomedical use [28]. Thus, in order to select CDs with homogeneous size distribution and specific surface functional groups, the product was purified by size exclusion chromatography (SEC). The use of SEC also allows to select the most red-emitting fractions of CDs with marked NIR-triggered photothermal property [26]. Atomic force microscopy (AFM) and size distribution obtained from the heights of the nanoparticles are reported in Figure 1a,a’ and show nanodots with average diameter of 1.5 nm, confirming the narrow size distribution that allow their potential biomedical applications. The selected CDs were characterized by hydroxyl, amine and amide surface groups and a high content of carboxyl group, as detected by FTIR spectroscopy (Figure 1b). The IR spectra show many diagnostic bands such as O-H stretching (3420 cm^−1^), N-H stretching (3200 cm^−1^), asymmetric (1711 cm^−1^) and symmetric COOH stretching (1381 cm^−1^), and the amide I band (1620 cm^−1^).

The CDs show different absorption bands in the UV/vis spectrum. Based on the absorption bands detected, the emission spectrum was recorded under excitation at 540 nm. The fluorescence spectra present a significant red emission band from 580 to 750 nm with a peak at 610 nm (Figure 1c).

The photoluminescence displayed by these CDs is of particular interest in theranostic applications, since an emission within the biological transparent window (red-NIR bands) is the minimum set of optical properties required to obtain high resolution images in fluorescence imaging [29]. Hence, the selected CDs are suitable excipients to produce PLGA nanoparticles with theranostic properties in view of imaging-guided anticancer therapies.

### 3.2. Preparation of PLGA-CDs Hybrid Nanoparticles and Their Characterization

The selected red-emitting CDs endowed with good photothermal properties were employed as functional excipient to obtain smart PLGA-CDs nanoparticles with fluorescence and photothermal features and high stability in aqueous media. The idea was to entrap CDs inside PLGA nanoparticles during the nanoprecipitation process, thus modifying their bulk physicochemical properties such as photoluminescence, photothermal sensitivity and water dispersibility. In fact, a proper amount of CDs might be sufficient to impinge on the surface properties, thus conferring suitable hydrophilicity owing to the presence of polar surface groups on the CDs surface. We prepared PLGA-CDs nanoparticles by solvent displacement technique by dripping an organic PLGA/CDs colloidal dispersion in a large excess of “non-solvent”. The rapid diffusion of the organic solvent in water allows the formation of well-dispersed nanoparticles. We obtained nanoparticles with low (1%) and high (20%) CDs content so as to study the effect of the CDs on the stability and optical properties. All the obtained formulations were analyzed with dynamic light scattering techniques in order to evaluate the size distribution and surface charge.

**Figure 2:**
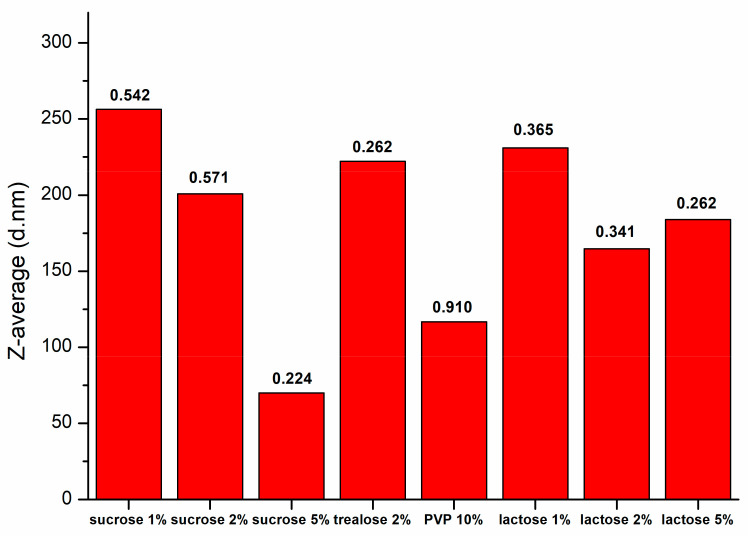
Mean size and PDI values of PLGA-CDs1% NPs with different cryoprotectant after lyophilization.

For the PLGA-CDs nanoparticles containing a low content of CDs (PLGA-CDs1%), unfortunately, we observed aggregation after the lyophilization process and redispersion was not allowed. Therefore, according with available literature data, the use of a cryoprotectant agents was required. Usually PLGA nanoparticles can be stabilized using a variety of cryoprotectant, including PVA, PVP and sucrose, at a concentration varying from 2 to 5% *w*/*v* [30,31,32]. In order to understand which type and concentration of cryoprotectant agent give the best redispersion of PLGA-CDs1% nanoparticles DLS measurements were performed after redispersion of freeze-dried samples containing different common cryoprotectors at different concentrations (Figure 2). The best result, in term of Z-average and PDI, was obtained with 5% sucrose (d = 70 nm; PDI = 0.224), while, using 10% *w*/*v* polyvinylpyrrolidone (PVP) an acceptable redispersion was obtained in terms of size, but with high polydispersity values (d = 116.6 nm; PDI = 0.910).

Differently, for the PLGA-CDs nanoparticles containing high amount of CDs (PLGA-CDs20%) we observed a slight higher diameter (from 54 to 74 nm) and a better redispersion after lyophilization, obtaining a colloidal dispersion easily stabilized using significantly lower amount of cryoprotectant (1% PVP) (Table 1). In fact, while the crude PLGA-CDs20% sample give rise to a slight aggregation after the lyophilization process (d = 291 nm, data not shown), adding only 1% of PVP in the PLGA-CDs20% sample we observed nanoparticles with comparable dimensions and polydispersity index of the starting sample (d = 93.2 nm; PDI = 0.181) (Table 1). The different redispersion observed between PLGA-CDs20% and PLGA-CDs1% nanoparticles can be ascribed to the different surface charge of the nanoparticles owing to the presence of CDs on the PLGA surface. Indeed, Zeta-potential measurements were performed on freshly prepared samples and show a quasi-neutral charge for PLGA-CDs1% nanoparticles (ζ = −5.7 ± 4.4), while for PLGA-CDs20% the Zeta-potential was quite negative (ζ = −30.3 ± 17.6), implying the presence of carboxyl groups of CDs on the surface responsible for the higher stability in aqueous media. This difference is due to the higher surface arrangement of CDs (negatively charged) in the PLGA-CDs20% nanoparticles respect to PLGA-CDs1% ones, where the amount of CDs in too low to provide a significant surface modification.

It should be noticed that irinotecan hydrochloride can affect the arrangement of drug loaded PLGA-CDs nanoparticles. Indeed, nanoparticles with hydrodynamic diameter three times bigger are formed in the presence of irinotecan for the PLGA-CDs1% and almost two times bigger for the PLGA-CDs20% (Table 1). This can be ascribed to strong interaction between irinotecan (cationic) and CDs (anionic) owing to the opposite charge possessed under the adopted conditions.

**Figure 3:**
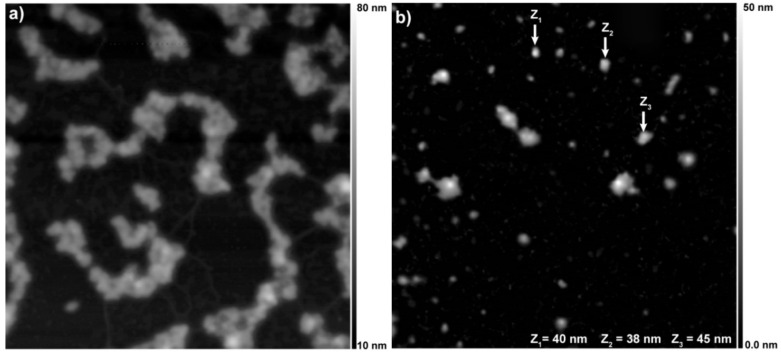
Atomic force microscopy of the PLGA-CDs1% (**a**) and PLGA-CDs20% (**b**) nanoparticles. Z represents the height of the nanoparticles. Observed field, 16 μm^2^ (4 × 4 μm).

The lower tendency of the PLGA-CDs20%NPs sample to form bigger aggregates was also confirmed by atomic force microscopy (AFM) studies. As shown in Figure 3a, PLGA-CDs1% NPs show a strong tendency to form aggregates after drying the aqueous dispersion on mica discs. It can be seen the formation of micrometric aggregates of nanoparticles of about 50 nm in diameter. Furthermore, there are not free CDs throughout the sample implying that it consists of nanocomposite CDs-embedded PLGA nanoparticles. Whilst, AFM micrographs obtained for the PLGA-CDs20% sample after evaporation of water do not highlight aggregates, but only well-defined nanoparticles with roughly 45–50 nm in diameter, thus confirming the role of CDs on the stabilization of nanoparticles in aqueous media (Figure 3b). AFM micrograph of the PLGA-CDs20% sample also provides evidence that there are not free CDs in the sample, indicating that the optical properties observed below can be ascribed to the CDs entrapped inside PLGA nanoparticles.

### 3.3. Optical and Photothermal Characterization of the PLGA-CDs Nanoparticles

**Figure 4:**
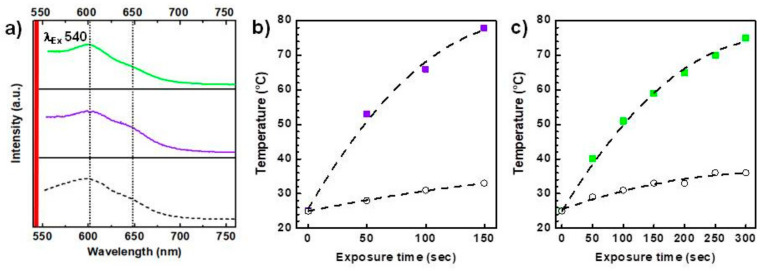
Emission spectra of CDs (black dash), PLGA-CDs20% NPs (purple solid) and PLGA-CDs1% NPs (green solid) in water (**a**). Photothermal kinetics of PLGA-CDs20% NPs (2.5 W cm^−2^) (**b**) and PLGA-CDs1%NPs (7 W cm^−2^) (**c**) (0.1 mg mL^−1^): ultrapure water was used as negative control (dark open symbol).

With the aim of understanding if they can be used in IG-PPT applications, the photothermal and red fluorescence properties of PLGA-CDs nanoparticles were established. The emission spectra under excitation at 540 nm of both nanoparticles were compared with that of plain CDs. As evidenced in Figure 4a both PLGA-CDs NPs spectra show the same characteristic emission band of the CDs in the red region. The increase of CDs content in nanoparticles from 1 to 20% is accompanied by changes of shape in the emission band. In detail, the shoulder at 650 nm is more pronounced in nanoparticles with 20% of CDs, similar to nude CDs. On the contrary, the lower content of CDs (1%) leads to a flattening of the emission bands. This phenomenon can be due to CDs-CDs interactions in the PLGA-CDs20% sample, which reflect surface electronic transitions responsible for red emission. On the whole, the emission intensity of the latter is much more intense than that observed for the PLGA-CDs1% nanoparticles, suggesting that these should be desired in fluorescence imaging applications.

The ability of both nanoparticles to convert NIR light into heat so as to act as active agents in IG-PPT was evaluated irradiating a dispersion of either a PLGA-CDs20% or PLGA-CDs1% dispersion in water and measuring the temperature increase by using an optical fiber. The conditions used for the two samples were different, since the higher power used for the PLGA-CDs1% sample provoked the overheating of the dispersion and the fusion of the nanoparticles. In particular, photothermal kinetics time were attained using an 810 nm laser diode source for 150 s with a power of 2.5 W cm^−2^ and for 300 s at 7 W cm^−2^ for the PLGA-CDs20% and PLGA-CDs1%NPs, respectively.

As show in Figure 4b,c a higher amount of CDs (20% on a weight basis) confers to the nanoparticles a sharper photothermal conversion capacity in comparison with the PLGA-CDs1% NPs. It might be noticed that this trend is corroborated by the lower potency used for the PLGA-CDs20% sample. In fact, three times lower potency was employed to obtain comparable photothermal results. In particular, the PLGA-CDs20% sample reaches the minimum temperature of hyperthermia (42 °C) [33] after about 30 s of laser treatment at 2.5 W cm^−2^, while the second one needs twice of the irradiation time at 7 W cm^−2^. Therefore, higher amount of CDs is required not only to obtain a good physicochemical stability in aqueous media, but also to prepare the PLGA-based nanoparticles with promising optical properties in terms of fluorescence and photothermal properties, needed for their application in IG-PTT.

### 3.4. Preparation of the Irinotecan-loaded PLGA-CDs Nanoparticles

The irinotecan-loaded nanoparticles, namely PLGA-CDs1%@IT and PLGA-CDs20%@IT, were prepared using the same protocol used for the empty PLGA-CDs samples, but adding to the PLGA/CDs colloidal dispersion an amount of irinotecan hydrochloride. The ability of PLGA-CDs NPs to entrap the drug was evaluated by HPLC analysis. Results show that the amount of CDs enclosed into nanoparticles have a relevant role in ensuring higher drug loading (DL). In fact, PLGA-CDs20% nanoparticles have a DL of 4.73 ± 0.15% on a weight basis, significantly higher if compared with the parent nanoparticles containing lower amount of CDs (DL of 3.5 ± 0.11%). Drug loaded nanoparticles where characterized in term of size distribution. For comparative purposes results are reported in Table 1. As shown the loading process has an impact on the size distribution of both samples and it is highly influenced by the presence of CDs inside nanoparticles. In particular, the virgin drug-loaded PLGA nanoparticles results too much big to be taken into consideration for this study (d > 600 nm, data not shown), while the presence of % CDs during the loading afford to nanoparticles with dimension about three times higher than that obtained for the PLGA-CDs1% sample (54 vs. 158 nm). For the PLGA-CDs20%@IT sample the diameter passed from 74 to 102 nm, suggesting that particle arrangement during the loading process is favorably influenced by the CDs dispersed throughout the PLGA matrix. No changes were observed after the lyophilization process for both PLGA-CDs1%@IT and PLGA-CDs20%@IT (Table 1).

### 3.5. Evaluation of Irinotecan Loading and Release from PLGA-CDs@IT Nanoparticles

The ability of the PLGA-CDs@IT nanoparticles of releasing their drug payload under physiological conditions was evaluated in PBS pH 7.4 using the dialysis method.

**Figure 5:**
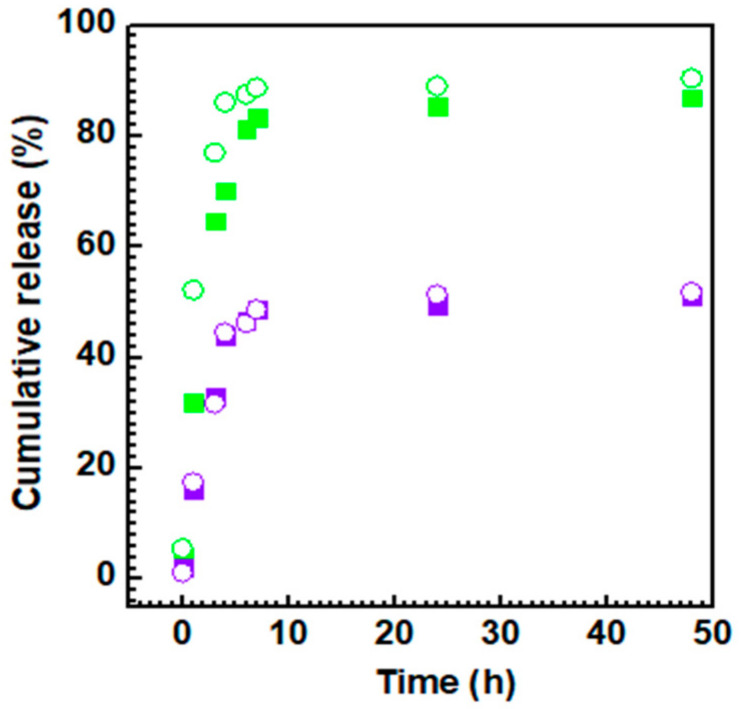
Cumulative drug release of PLGA-CDs1% NPs (green solid square) and PLGA-CDs20% NPs (violet solid square). Drug release after laser irradiation of PLGA-CDs1% NPs (green open circle) and PLGA-CDs20% NPs (violet open circle).

The cumulative release kinetics obtained are reported in Figure 5. It can be seen that as for the other experiments the amount of CDs in the PLGA-CDs nanoparticles have a great impact in the release kinetic observed. In particular, PLGA-CDs1%@IT released approximately all the irinotecan payload in 10 h, similar to the kinetic release of PLGA nanoparticles reported in literature [34]. On the contrary, PLGA-CDs20%@IT released only 50% of its payload in 48 h, displaying a prolonged and sustained release capability without hinting at a burst effect. As widely reported in literature, the NIR-triggered photothermal effect of nanoheaters as CDs, allows a faster drug release due to an increase in temperature [8,35]. However, the drug release kinetics of both PLGA-CDs NPs after laser irradiation were comparable to the kinetics obtained by untreated nanoparticles. It is possible to hypothesize that the failed burst drug release following laser treatment is due to the inability of the nanosystem to disassemble in a temperature-dependent fashion. As consequence, being the photothermal and photoluminescence behavior superior and showing higher stability and sustained drug release profile, the PLGA-CDs20%@IT sample was selected as a candidate for the study of anticancer efficacy and bioimaging.

### 3.6. Biological Characterization of PLGA-CDs20%@IT NPs

**Figure 6:**
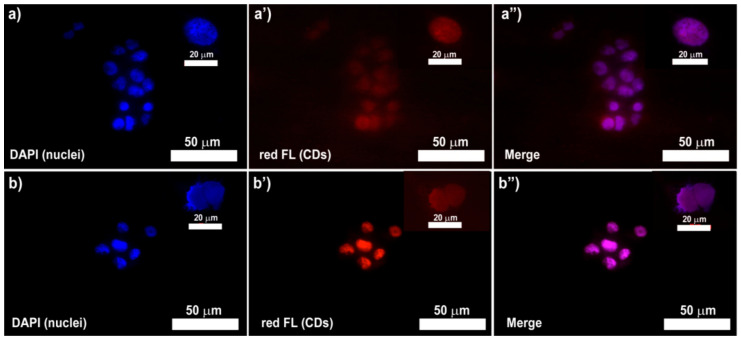
Uptake of PLGA-CDs20% NPs on human breast cancer cell culture (MDA-MB-231) after 6 h (**a**–**a”**) and 24 h (**b**–**b”**) of incubation. Magnification 40× (insert: 100×).

To demonstrate the ability of PLGA-CDs20% NPs of acting as contrast agent in FLI, fluorescence microscopy (λ_Ex_ = 559) was employed to track the nanosystem in living breast cancer cells (MDA-MB-231) after 6 and 24 h of incubation (Figure 6). The PLGA-CDs20% nanoparticles show excellent contrast in FLI once internalized inside cancer cells. They can be tracked using red fluorescence channel just after 6 h from the incubation and appear prevailingly localized inside MDA-MB-231 nuclei (Figure 6a–a”). Red fluorescence stands out after 24 h of incubation and appear much more marked, implying that cell internalization in MDA-MB-231 is time-dependent and efficient (Figure 6b–b”).

However, considering that the higher nuclear pore size is about 10 nm and the diameter of the nanoparticles is about 100 nm, they may not be localized into cell nuclei as they are. Therefore, it is possible to hypothesize that the intracellular microenvironment allows the disassembly of the nanoparticles giving rise CDs release and diffusion inside nuclei.

**Figure 7:**
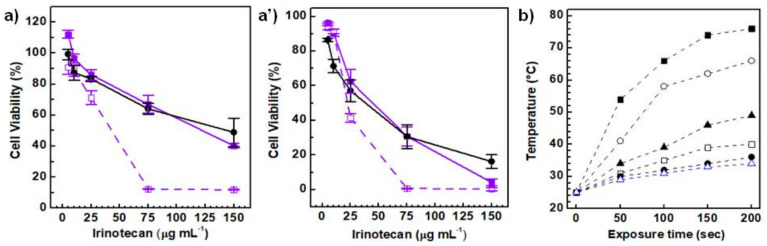
Anticancer activity on MDA-MB-231 after 24 h and 48 h of incubation (**a**,**a’**): irinotecan (dark solid line, close circle), PLGA-CDs20%@IT NPs (violet solid line, solid square), PLGA-CDs20% NPs + laser (violet dash line, open square). Photothermal effect of the PLGA-CDs20% NPs dispersion in DMEM at different concentration ranging from 5 to 150 μg mL^−1^. DMEM was used as control (blue open triangle) (**b**).

The in vitro cytotoxic effects of the PLGA-CDs20%@IT NPs was carried out on the MDA-MB-231 cell line after 24 and 48 h of incubation (Figure 7a,a’). A time-dependent and dose-dependent efficacy was observed as cell viability decreased with the incubation time and increasing the amount of irinotecan.

CDs are efficient NIR-photothermal agents due to their high photothermal conversion property within the biological transparency window, needed to induce cancer cell death by photothermal ablation [6]. Hence, in a second set of experiments it was evaluated the NIR-triggered photothermal ablation of cancer cells (MDA-MB-231), in combination with the cytotoxic effect induced by the drug released in situ (Figure 7a,a’). The PLGA-CDs20%@IT nanoparticles were incubated for 24 and 48 h, and then treated with an 810 nm laser diode for 100 s with power fitted at 2.5 W cm^−2^. The combined cytotoxic effects were expressed as reduction of cell viability with respect with the untreated control. As shown in Figure 7a, the difference in term of cell viability observed between cells treated with the laser and the untreated ones is more marked just after 24 h of incubation. The photothermal treatment seems also much more effective if compared with the free drug for all the concentration range considered. A similar trend was observed for the cells treated after 48 h of incubation (Figure 7a’).

The effective ablation of cancer cells observed for the cells treated with concentration of PLGA-CDs20%@IT higher than 75 μg mL^−1^ can be ascribed to the extremely high temperatures reached inside wells during the photothermal experiments. Indeed, we measured the temperature for each well in a parallel experiment in which the hyperthermic effect was assessed on dispersions of PLGA-CDs20% NPs in DMEM at the same conditions used for the in vitro cytotoxic studies (5–150 μg mL^−1^). The ability of the nanosystem to convert the laser energy source into heat was investigated applying a 810 nm laser for 200 s, measuring the dispersion temperature variation at scheduled time intervals. As shown in Figure 7b, the heating rate is higher as the concentration of the PLGA-CDs20% NPs increases. In particular, at concentration higher than 75 μg mL^−1^ and after 200 s of exposure time the well temperature overtakes 60 °C (Figure 7b), explaining well why cells are totally ablated under these conditions (Figure 7a,a’).

It is worthy of remark that the IC_50_ and maximum inhibition (I_max_) values reported in Table 2 highlight the higher potency of the PLGA-CDs20%@IT NPs even if compared with the free drug. The IC_50_ values of the PLGA-CDs20%@IT NPs triggered with the laser, both after 24 and 48 h of incubation, were about half of the values obtained from the drug-loaded nanosystem without the laser treatment and lower than that obtained for irinotecan alone. The I_max_ values were about two times higher than that observed for both cells treated with the free drug and the PLGA-CDs20%@IT NPs after 24 h of incubation, suggesting a greater efficacy of the photothermal treatments. On the whole, cell viability data and IC_50_ and I_max_ values confirm the potent anticancer effect of the theranostic approach proposed, combining chemotherapy and photothermal therapy in a unique biodegradable and bioeliminable platform consisting of PLGA and CDs.

## 4. Conclusions

In the present work, hybrid nanoparticles (PLGA-CDs) consisting of red-emitting carbon-based nano-heaters embedded into a poly(lactic-*co*-glycolic acid) matrix were developed. PLGA-CDs nanoparticles are designed to display in a single biodegradable and bioeliminable nanoplatform drug delivery, photothermal and imaging abilities useful in anticancer imaging-guided photothermal therapy. Herein, for the firt time the role of CDs as multifunctional excipient is studied as function of the concentration inside PLGA nanoparticles. We demonstrated that the presence of CDs inside the biodegradable PLGA nanomatrix impinge on several formulation parameters determining the stability in physiological fluids, drug loading processes and drug release profiles. Although PLGA allowed an increase in size, reducing premature renal excretion of CDs, the encapsulation of CDs proved capable of effectively stabilizing the system, also increasing drug loading capacity and prolonging the release over time. The stabilizing role of CDs was demonstrated by DLS and AFM data by comparing two hybrid nanosystems with different CDs content (PLGA-CDs1% and PLGA-CDs20%), and showing that CDs avoid aggregation at the higher concentration used by modifying surface properties of PLGA nanoparticles. PLGA-CDs20% NPs showed good in vitro bioimaging capabilities, as demonstrated by the marked red fluorescence observed by fluorescence microscopy on MDA-MB-231 cells treated with nanoparticles. Furthermore, the use of the PLGA-CDs20% in photothermal therapy combined with a suitable drug release was found to be effective in inducing cell death, as evaluated by cytotoxicity studies on MDA-MB-231 breast cancer cell cultures. Therefore, taking into account the ability of PLGA-CDs20% NPs of combining high water stability, red fluorescence imaging, targeted chemotherapy and NIR-triggered photothermal therapy, the use of CDs as functional excipient is a promising strategy in the formulation of nanoparticles for anticancer IG-PTT.

## Figures and Tables

**Table 1:** Summary size and PDI of PLGA-CDs NPs and PLGA-CDs@IT NPs pre- and post-lyophilization.

Samples	Pre Lyophilization		Post Lyophilization (with Cryoprotectant)	
	Z-Average (d-nm)	PDI	Z-Average (d-nm)	PDI
PLGA-CDs1%	54.78	0.117	70 *	0.224 *
PLGA-CDs20%	74.36	0.167	93.2 **	0.181 **
PLGA-CDs1%@IT	157.9	0.024	181.2 *	0.067 *
PLGA-CDs20%@IT	102.5	0.111	133.9 **	0.120 **

* cryoprotectant: 5% sucrose; ** cryoprotectant: 1% PVP 40 kDa.

**Table 2:** Half minimal inhibitory concentration (IC_50_) and maximal inhibition (I_max_) of PLGA-CDs20%@IT NPs compared to the free drug (IT) after 24 and 48 h of incubation on 2-D culture of MDA-MB-231.

Samples	IC_50_^24 h^ (μg mL^−1^)	IC_50_^48 h^ (μg mL^−1^)	I_max_^24 h^ (%)	I_max_^48 h^ (%)
Irinotecan	143.76	38	51.18	83.75
PLGA-CDs20%@IT NPs	121.12	45.10	59.80	95.79
PLGA-CDs20%@IT NPs + laser	43.14	22.30	88.15	99.69
